# Effects of Interobserver Variability on 2D and 3D CT- and MRI-Based Texture Feature Reproducibility of Cartilaginous Bone Tumors

**DOI:** 10.1007/s10278-021-00498-3

**Published:** 2021-08-17

**Authors:** Salvatore Gitto, Renato Cuocolo, Ilaria Emili, Laura Tofanelli, Vito Chianca, Domenico Albano, Carmelo Messina, Massimo Imbriaco, Luca Maria Sconfienza

**Affiliations:** 1 Dipartimento Di Scienze Biomediche Per La Salute, Università Degli Studi Di Milano, Via Luigi Mangiagalli 31, 20133 Milan, Italy; 2 Dipartimento Di Medicina Clinica E Chirurgia, Università Degli Studi Di Napoli “Federico II”, Naples, Italy; 3 Laboratory of Augmented Reality for Health Monitoring (ARHeMLab), Dipartimento Di Ingegneria Elettrica E Delle Tecnologie Dell’Informazione, Università Degli Studi Di Napoli “Federico II”, Naples, Italy; 4 Unità di Radiodiagnostica, Presidio CTO, ASST Pini-CTO, Milan, Italy; 5 Dipartimento di Radiologia Diagnostica ed Interventistica, Università degli Studi di Milano, Ospedale San Paolo, Milan, Italy; 6 Ospedale Evangelico Betania, Naples, Italy; 7 Clinica Di Radiologia, Istituto Imaging Della Svizzera Italiana - Ente Ospedaliero Cantonale, Lugano, Switzerland; 8 IRCCS Istituto Ortopedico Galeazzi, Milan, Italy; 9 Sezione Di Scienze Radiologiche, Dipartimento Di Biomedicina, Neuroscienze E Diagnostica Avanzata, Università Degli Studi Di Palermo, Palermo, Italy; 10 Dipartimento Di Scienze Biomediche Avanzate, Università Degli Studi Di Napoli “Federico II”, Naples, Italy

**Keywords:** Artificial intelligence, Chondroma, Chondrosarcoma, Neoplasms, Radiomics, Texture analysis

## Abstract

This study aims to investigate the influence of interobserver manual segmentation variability on the reproducibility of 2D and 3D unenhanced computed tomography (CT)- and magnetic resonance imaging (MRI)-based texture analysis. Thirty patients with cartilaginous bone tumors (10 enchondromas, 10 atypical cartilaginous tumors, 10 chondrosarcomas) were retrospectively included. Three radiologists independently performed manual contour-focused segmentation on unenhanced CT and T1-weighted and T2-weighted MRI by drawing both a 2D region of interest (ROI) on the slice showing the largest tumor area and a 3D ROI including the whole tumor volume. Additionally, a marginal erosion was applied to both 2D and 3D segmentations to evaluate the influence of segmentation margins. A total of 783 and 1132 features were extracted from original and filtered 2D and 3D images, respectively. Intraclass correlation coefficient ≥ 0.75 defined feature stability. In 2D vs. 3D contour-focused segmentation, the rates of stable features were 74.71% vs. 86.57% (*p* < 0.001), 77.14% vs. 80.04% (*p* = 0.142), and 95.66% vs. 94.97% (*p* = 0.554) for CT and T1-weighted and T2-weighted images, respectively. Margin shrinkage did not improve 2D (*p* = 0.343) and performed worse than 3D (*p* < 0.001) contour-focused segmentation in terms of feature stability. In 2D vs. 3D contour-focused segmentation, matching stable features derived from CT and MRI were 65.8% vs. 68.7% (*p* = 0.191), and those derived from T1-weighted and T2-weighted images were 76.0% vs. 78.2% (*p* = 0.285). 2D and 3D radiomic features of cartilaginous bone tumors extracted from unenhanced CT and MRI are reproducible, although some degree of interobserver segmentation variability highlights the need for reliability analysis in future studies.

## Introduction

Cartilaginous tumors of the bone include a broad spectrum of lesions that range from benign to malignant entities [1, 2]. Reliable identification and grading are crucial, as clinical management varies widely. Specifically, asymptomatic benign enchondromas do not require any treatment, appendicular atypical cartilaginous tumors are managed with intralesional curettage or even watchful waiting, and appendicular higher grade lesions and axial skeleton chondrosarcomas are resected with free margins [3]. The diagnosis relies on a combination of clinical presentation, imaging, and biopsy [3, 4]. Imaging, and particularly magnetic resonance imaging (MRI), has good accuracy in discriminating atypical cartilaginous tumors from higher grade lesions [5] but is less reliable in differentiating the former from enchondromas [6]. Biopsy is considered the reference standard but has the disadvantages of sampling errors [7] and discrepancies even among specialized bone pathologists due to overlapping histological findings [8]. Additionally, the risk of biopsy-tract contamination remains a concern. Thus, the need for cutting-edge imaging-based tools, such as radiomics, is advocated to safely diagnose and grade cartilaginous bone tumors non-invasively [9].

Texture analysis is a post-processing method for quantification of tumor heterogeneity, which reflects adverse tumor biology but cannot be captured using conventional imaging modalities or sampling biopsies [10]. It belongs to the growing field of radiomics, which includes extraction, analysis, and interpretation of large amounts of quantitative parameters from medical images [11, 12]. To date, texture analysis has been used to discriminate tumor grades and types before treatment, monitor response to therapy, and predict outcome [13]. The resulting quantitative parameters, known as texture or radiomic features, may suffer however from interobserver variability, particularly with regard to tumor delineation while performing manual segmentation [14–16]. The influence of segmentation margins is also critical because of textural details of the peritumoral area, which may affect the reproducibility of texture features and therefore their diagnostic performance [17]. In literature, the intraclass correlation coefficient (ICC) is commonly employed to assess radiomic feature reproducibility [17–21].

The aim of this study is to investigate the influence of interobserver manual segmentation variability on the reproducibility of bidimensional (2D) and volumetric (3D) unenhanced computed tomography (CT)- and MRI-based texture analysis in cartilaginous bone tumors.

## Materials and Methods

### Design and Population

The local Institutional Review Board approved this retrospective study and waived the need for informed consent. According to the ICC guidelines by Koo et al. [22], we designed our study to meet the numerical requirements of a reliability analysis in terms of both patients and observers involved, namely 30 lesions and 3 different readers [22]. A search of the radiology information system was performed and 30 patients with cartilaginous bone tumors were recruited (median age 52 [range, 28–72] years), including 10 enchondromas, 10 atypical cartilaginous tumors, and 10 chondrosarcomas. Inclusion criteria were as follows: (i) enchondromas proven either by histology or minimum follow-up of 6 years without alteration in shape or size and typical imaging findings of lobulated morphology and T2-weighted hyperintensity on MRI; (ii) histology-proven atypical cartilaginous tumors; (iii) histology-proven primary conventional grades II–III or dedifferentiated chondrosarcomas; (iv) 1.5-T MRI including turbo spin echo T1-weighted and T2-weighted sequences and 64-slice CT performed within 1 month before biopsy, intralesional curettage, or surgical resection for tumors diagnosed by histology. Exclusion criteria were the presence of pathological fracture and ambiguous histology report.

Enchondromas were located in the femur (*n* = 5), fibula (*n* = 2), foot phalanx (*n* = 1), humerus (*n* = 1), and radius (*n* = 1); atypical cartilaginous tumors in the femur (*n* = 2), fibula (*n* = 2), and humerus (*n* = 6); chondrosarcomas in the calcaneus (*n* = 1), femur (*n* = 2), humerus (*n* = 1), pelvis (*n* = 2), spine (*n* = 3), and tibia (*n* = 1).

### Image Segmentation

**Fig. 1:**
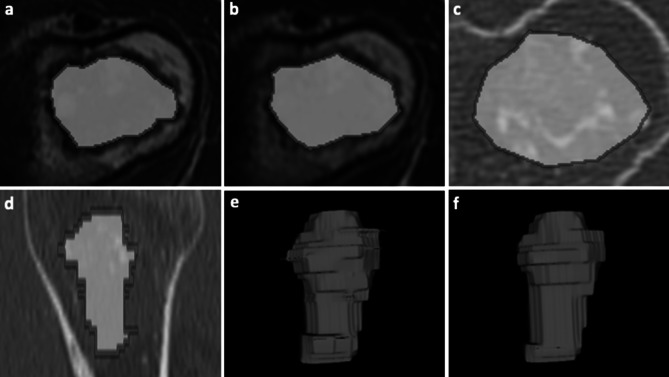
Contour-focused and margin shrinkage segmentation of an atypical cartilaginous tumor of the humerus in a 45-year-old woman. **a**–**c** 2D contour-focused segmentation was performed on axial T1-weighted MRI **a**, T2-weighted MRI **b**, and bone-window CT **c** on the slice showing the largest tumor extension. **d** 3D contour-focused segmentation was performed slice by slice in the axial plane to include the whole tumor volume, as shown in the sagittal CT image. Contour-focused segmentation provided the ROI including both green and red areas. Margin shrinkage segmentation provided the ROI including only the green area by computing a marginal erosion, which is shown in red. **e**–**f** Segmented tumor volumes obtained with 3D contour-focused **e** and margin shrinkage **f** segmentation are shown, where the latter has smoother margins as a result of marginal erosion

A musculoskeletal radiologist (S.G.) and two last-year radiology residents trained in musculoskeletal and oncologic imaging (I.E. and L.T.) independently performed manual image segmentation using the open-source software ITK-SNAP (v3.6) [23]. The readers knew the study would deal with cartilaginous bone tumors, but they were blinded to any other information regarding histological grade, disease course, and additional imaging studies. All tumors were segmented on axial CT scans and on axial MRI sequences as first choice and coronal or sagittal sequences as second choice. Manual contour-focused segmentation was performed on unenhanced bone-window CT and T1-weighted and T2-weighted MRI by drawing both a 2D region of interest (ROI) on the slice showing the largest tumor area and a 3D ROI including the whole tumor volume. The “polygon mode” ITK-SNAP tool was used for all segmentations. While segmenting the tumors on CT, the readers used the MRI sequences to aid contour identification of each tumor. Thereafter, margin shrinkage segmentation was computed by applying a marginal erosion to both 2D and 3D segmentations in order to evaluate the influence of segmentation margins on feature reproducibility (Fig. 1). In detail, ROI shrinkage was performed using the fslmaths erosion function of the FMRIB Software Library [24]. The default 2D and 3D kernels, which are 3 × 3 × 1 and 3 × 3 × 3 boxes centered at the target voxel, were employed as appropriate. During the erosion process, each voxel in the ROI is targeted sequentially, and its value is changed to 0 (i.e., removed from the ROI) if a zero-value voxel is found within the kernel. Therefore, the shrinkage was usually more extensive for 3D ROIs compared to 2D ones.

### Texture Analysis

Image pre-processing consisted in resampling to a 2 × 2 isotropic pixel or 2 × 2 × 2 isotropic voxel, whole-image intensity normalization (mean value of 300 and standard deviation of 100), and discretization with a fixed bin width of 5. Original CT and MRI and 2D and 3D ROIs were used for feature extraction on PyRadiomics (v2.2.0) [25], an open-source Python software. The extracted features were grouped according to PyRadiomics official documentation (https://pyradiomics.readthedocs.io/en/latest/features.html), as follows:

- 18 first-order features, which describe the distribution of pixel or voxel gray-level values;
- 9 shape-based 2D and 14 shape-based 3D features, which respectively describe the 2D and 3D size and shape of the ROI;
- 22 Gy-level cooccurrence matrix (GLCM) features, which quantify how often pairs of pixels or voxels with certain values occur in a specified spatial range;
- 16 Gy-level size zone matrix (GLSZM) features, which quantify gray-level zones, i.e., the number of connected pixels or voxels sharing the same gray-level value;
- 16 Gy-level run length matrix (GLRLM) features, which quantify gray-level runs, i.e., the length in number of consecutive pixels or voxels having the same gray-level value;
- 14 Gy-level dependence matrix (GLDM) features, which quantify gray-level dependencies, i.e., the number of connected pixels or voxels within a set distance that are dependent on the center pixel and voxel.

In addition to the original CT and MRI, Laplacian of Gaussian (LoG)-filtered (sigma = 2, 3, 4, 5) and wavelet-transformed 2D and 3D images (all possible low- and high-pass filter combinations) were obtained for extraction of first-order and matrix features. Shape-based features are independent from gray-level value distribution and therefore were only computed on the original images. A total of 783 and 1132 features were extracted from original, LoG-filtered, and wavelet-transformed 2D and 3D images, respectively.

### Statistical Analysis

Texture feature interobserver reliability was assessed using a two-way, random-effects, single-rater, absolute agreement ICC. Features were considered stable when achieving good (0.75 ≤ ICC < 0.9) to excellent (ICC ≥ 0.9) interobserver reliability [22]. Differences among variables were evaluated using Chi-square test. A 2-sided *p*-value < 0.05 indicated statistical significance [26]. Data analysis was performed using the pandas and numpy Python software and the “irr” R package [27, 28].

### Machine Learning Analysis

To assess the potential value of CT and MRI texture features extracted from 2D and 3D annotations, an exploratory data analysis was performed with an Extra Trees (ET) ensemble model. The same pipeline was employed on all available datasets, consisting of feature selection through cross-validated recursive feature elimination (RFE) and random search hyperparameter tuning nested within a leave-one-out cross-validation on the entire dataset. RFE was conducted using tenfold cross-validation and an ET estimator with default hyperparameters. Then, in the training folds of the leave-one-out cross-validation, the synthetic oversampling technique was applied to balance the 3 classes (i.e., creating a synthetic instance to substitute the lesion in the test fold), followed by 100 iterations of ET hyperparameter random search. Given the presence of 3 classes with balanced cases, accuracy was used as the reference score for both RFE and ET tuning. The hyperparameter search space was as follows:

1. Number of trees = 100–1000
2. Criterion = entropy or Gini
3. Max depth = 1–10
4. Bootstrap = True or False
5. Max samples = 0–100%

## Results

In 2D contour-focused vs. margin shrinkage segmentation, the stable feature rates were 74.71% (*n* = 585) vs. 71.65% (*n* = 561), 77.14% (*n* = 604) vs. 76.12% (*n* = 596), and 95.66% (*n* = 749) vs. 96.42% (*n* = 755) for CT and T1-weighted and T2-weighted images, respectively. The number of stable features derived from 2D contour-focused segmentation showed no difference in comparison with 2D margin shrinkage segmentation (*p* = 0.343). Table 1 details the number and percentage of stable features that were obtained with 2D contour-focused segmentation, grouped according to feature class and image type.

**Table 1:** 2D contour-focused segmentation. Number and percentage of stable features with good (0.75 ≤ ICC < 0.9) and excellent (ICC ≥ 0.9) interobserver reliability grouped according to feature class and image type. *GLCM*, gray-level cooccurrence matrix; *GLDM*, gray-level dependence matrix; *GLRLM*, gray-level run length matrix; *GLSZM*, gray-level size zone matrix; *ICC*, intraclass correlation coefficient; *LoG*, Laplacian of Gaussian

**2D**	**Feature class**	**Image type**	**Total features (*n*)**	**ICC ≥ 0.75 (*n*)**	**ICC ≥ 0.75 (%)**	**ICC ≥ 0.90 (*n*)**	**ICC ≥ 0.90 (%)**
**CT**	First order	LoG	72	63	87.50	29	40.28
		Original	18	16	88.89	8	44.44
		Wavelet	72	53	73.61	20	27.78
	GLCM	LoG	88	78	88.64	31	35.23
		Original	22	13	59.09	5	22.73
		Wavelet	88	60	68.18	27	30.68
	GLDM	LoG	56	49	87.50	18	32.14
		Original	14	10	71.43	2	14.29
		Wavelet	56	34	60.71	10	17.86
	GLRLM	LoG	64	58	90.63	27	42.19
		Original	16	13	81.25	2	12.50
		Wavelet	64	43	67.19	12	18.75
	GLSZM	LoG	64	46	71.88	20	31.25
		Original	16	9	56.25	3	18.75
		Wavelet	64	32	50.00	14	21.88
	Shape	Original	9	8	88.89	7	77.78
	Overall		783	585	74.71	235	30.01
**T1w**	First order	LoG	72	65	90.28	42	58.33
		Original	18	15	83.33	8	44.44
		Wavelet	72	52	72.22	27	37.50
	GLCM	LoG	88	81	92.05	48	54.55
		Original	22	17	77.27	10	45.45
		Wavelet	88	67	76.14	50	56.82
	GLDM	LoG	56	43	76.79	29	51.79
		Original	14	10	71.43	7	50.00
		Wavelet	56	38	67.86	30	53.57
	GLRLM	LoG	64	51	79.69	34	53.13
		Original	16	12	75.00	9	56.25
		Wavelet	64	46	71.88	35	54.69
	GLSZM	LoG	64	50	78.13	26	40.63
		Original	16	8	50.00	6	37.50
		Wavelet	64	40	62.50	19	29.69
	Shape	Original	9	9	100.00	8	88.89
	Overall		783	604	77.14	388	49.55
**T2w**	First order	LoG	72	68	94.44	61	84.72
		Original	18	16	88.89	15	83.33
		Wavelet	72	60	83.33	48	66.67
	GLCM	LoG	88	86	97.73	79	89.77
		Original	22	22	100.00	18	81.82
		Wavelet	88	84	95.45	71	80.68
	GLDM	LoG	56	56	100.00	48	85.71
		Original	14	12	85.71	10	71.43
		Wavelet	56	53	94.64	31	55.36
	GLRLM	LoG	64	64	100.00	60	93.75
		Original	16	15	93.75	13	81.25
		Wavelet	64	63	98.44	45	70.31
	GLSZM	LoG	64	64	100.00	47	73.44
		Original	16	15	93.75	12	75.00
		Wavelet	64	62	96.88	41	64.06
	Shape	Original	9	9	100.00	8	88.89
	Overall		783	749	95.66	607	77.52

In 3D contour-focused vs. margin shrinkage segmentation, the stable feature rates were 86.57% (*n* = 980) vs. 83.66% (*n* = 947), 80.04% (*n* = 906) vs. 71.47% (*n* = 809), and 94.97% (*n* = 1075) vs. 65.72% (*n* = 744) for CT and T1-weighted and T2-weighted images, respectively. The number of stable features derived from 3D contour-focused segmentation was higher compared to 3D margin shrinkage segmentation (*p* < 0.001). Table 2 details the number and percentage of stable features that were obtained with 3D contour-focused segmentation, grouped according to feature class and image type.

**Table 2:** 3D contour-focused segmentation. Number and percentage of stable features with good (0.75 ≤ ICC < 0.9) and excellent (ICC ≥ 0.9) interobserver reliability grouped according to feature class and image type. *GLCM*, gray-level cooccurrence matrix; *GLDM*, gray-level dependence matrix; *GLRLM*, gray-level run length matrix; *GLSZM*, gray-level size zone matrix; *ICC*, intraclass correlation coefficient; *LoG*, Laplacian of Gaussian

**3D**	**Feature class**	**Image type**	**Total features (*n*)**	**ICC ≥ 0.75 (*n*)**	**ICC ≥ 0.75 (%)**	**ICC ≥ 0.90 (*n*)**	**ICC ≥ 0.90 (%)**
**CT**	First order	LoG	72	64	88.89	44	61.11
		Original	18	14	77.78	9	50.00
		Wavelet	144	114	79.17	93	64.58
	GLCM	LoG	88	86	97.73	65	73.86
		Original	22	22	100.00	19	86.36
		Wavelet	176	169	96.02	153	86.93
	GLDM	LoG	56	50	89.29	24	42.86
		Original	14	13	92.86	8	57.14
		Wavelet	112	98	87.50	71	63.39
	GLRLM	LoG	64	62	96.88	30	46.88
		Original	16	14	87.50	9	56.25
		Wavelet	128	112	87.50	86	67.19
	GLSZM	LoG	64	46	71.88	19	29.69
		Original	16	11	68.75	2	12.50
		Wavelet	128	93	72.66	67	52.34
	Shape	Original	14	12	85.71	7	50.00
	Overall		1132	980	86.57	706	62.37
**T1w**	First order	LoG	72	67	93.06	43	59.72
		Original	18	12	66.67	7	38.89
		Wavelet	144	121	84.03	89	61.81
	GLCM	LoG	88	77	87.50	47	53.41
		Original	22	16	72.73	10	45.45
		Wavelet	176	151	85.80	125	71.02
	GLDM	LoG	56	42	75.00	24	42.86
		Original	14	9	64.29	7	50.00
		Wavelet	112	85	75.89	60	53.57
	GLRLM	LoG	64	50	78.13	31	48.44
		Original	16	9	56.25	6	37.50
		Wavelet	128	99	77.34	77	60.16
	GLSZM	LoG	64	47	73.44	21	32.81
		Original	16	10	62.50	5	31.25
		Wavelet	128	97	75.78	55	42.97
	Shape	Original	14	14	100.00	11	78.57
	Overall		1132	906	80.04	618	54.59
**T2w**	First order	LoG	72	70	97.22	53	73.61
		Original	18	17	94.44	11	61.11
		Wavelet	144	126	87.50	94	65.28
	GLCM	LoG	88	81	92.05	69	78.41
		Original	22	21	95.45	15	68.18
		Wavelet	176	169	96.02	145	82.39
	GLDM	LoG	56	55	98.21	41	73.21
		Original	14	14	100.00	9	64.29
		Wavelet	112	106	94.64	73	65.18
	GLRLM	LoG	64	64	100.00	53	82.81
		Original	16	16	100.00	11	68.75
		Wavelet	128	122	95.31	92	71.88
	GLSZM	LoG	64	62	96.88	46	71.88
		Original	16	16	100.00	11	68.75
		Wavelet	128	122	95.31	70	54.69
	Shape	Original	14	14	100.00	9	64.29
	Overall		1132	1075	94.97	802	70.85

**Fig. 2:**
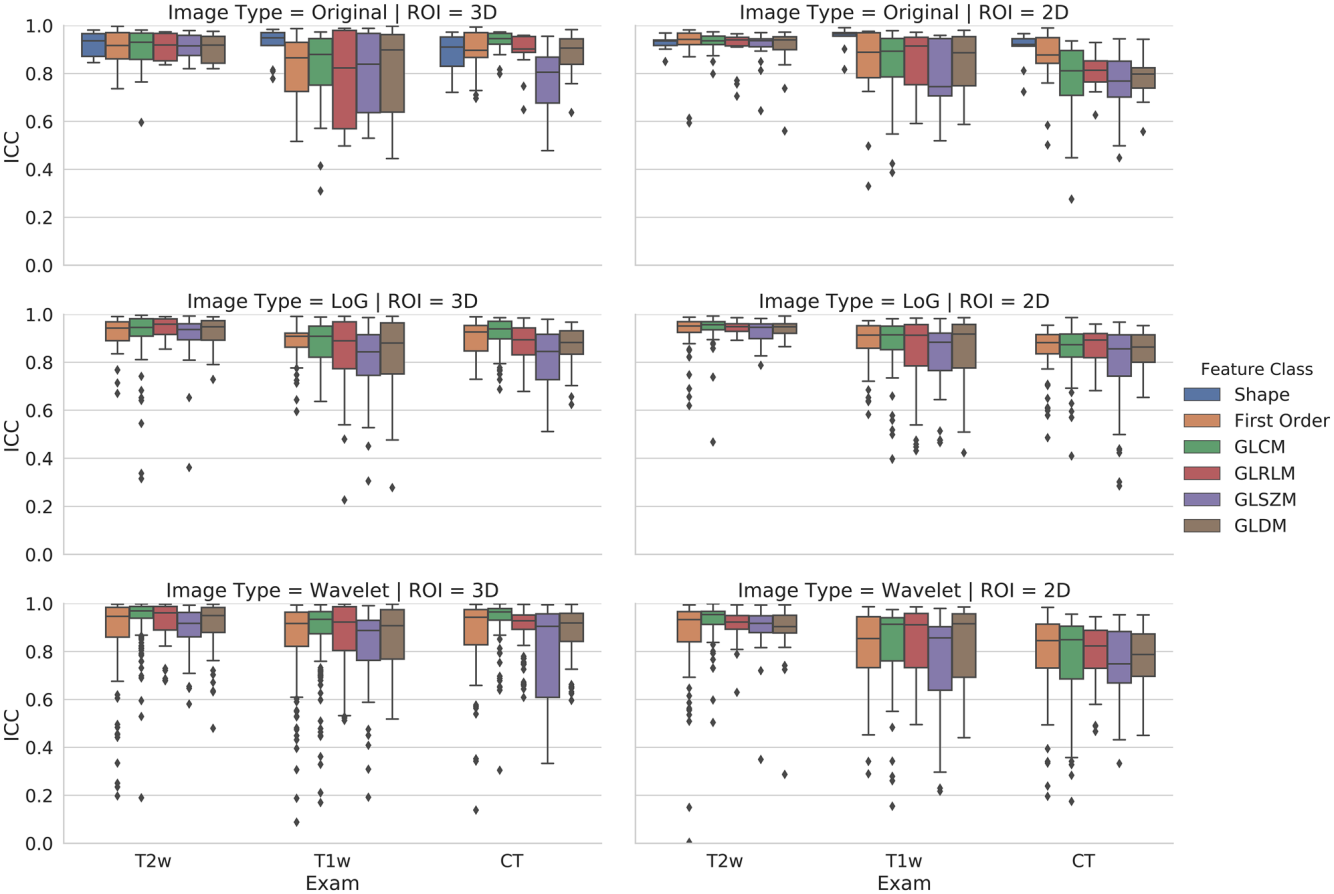
3D and 2D contour-focused segmentation. Box and whisker plots show the interobserver reliability of feature classes grouped according to image type

The rate of stable features derived from CT was higher for 3D compared to 2D contour-focused segmentation (*p* < 0.001), while no difference was found for features derived from T1-weighted and T2-weighted MRI between 3D and 2D contour-focused segmentation (*p* = 0.142 and 0.554, respectively). In Fig. 2, box and whisker plots show the interobserver reliability of feature classes derived from 3D and 2D contour-focused segmentation, grouped according to image type.

**Fig. 3:**
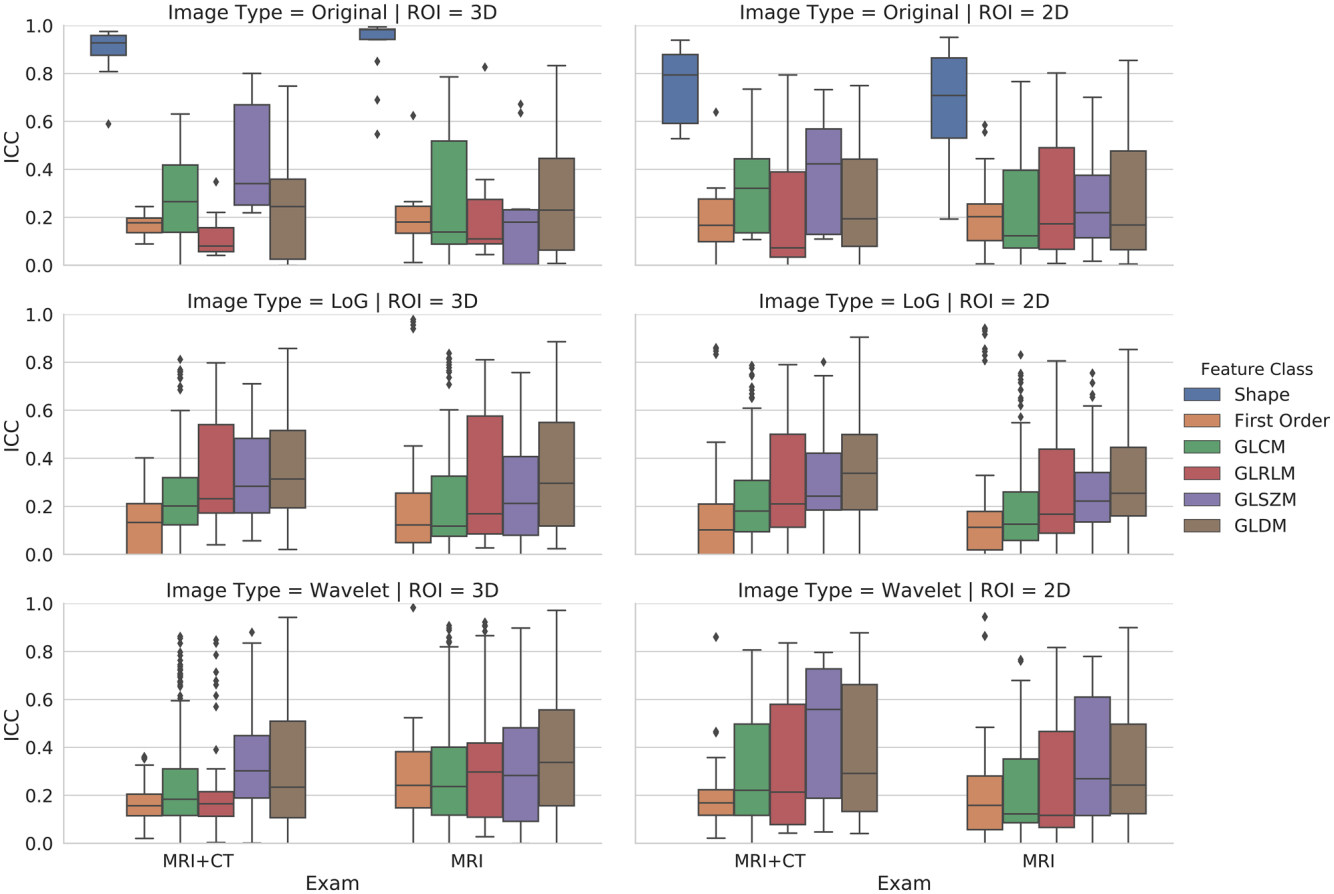
3D and 2D contour-focused segmentation. Box and whisker plots show the overall interobserver reliability of matching feature classes derived from CT and MRI, as well as T1-weighted and T2-weighted MRI sequences, grouped according to image type

In 2D vs. 3D contour-focused segmentation, matching stable features derived from CT and MRI were 65.77% (*n* = 515) vs. 68.73% (*n* = 778), and those derived from T1-weighted and T2-weighted images were 75.99% (*n* = 595) vs. 78.18% (*n* = 885), respectively (*p* = 0.191 and 0.285). Tables 3 and 4 respectively detail the number and percentage of matching stable features obtained with 2D and 3D contour-focused segmentation, as well as overall interobserver reliability across different imaging modalities and MRI sequences, grouped according to feature class and image type. In Fig. 3, box and whisker plots show the overall interobserver reliability of matching feature classes derived 3D and 2D contour-focused segmentation of CT and MRI, as well as MRI including T1-weighted and T2-weighted sequences, grouped according to image type. Most shape-based 2D and 3D features were stable even across different imaging modalities and MRI sequences.

**Table 3:** 2D matching features. Number and percentage of matching stable features obtained with 2D contour-focused segmentation, as well as number and percentage of matching stable features with good (ICC ≥ 0.75) overall interobserver reliability across different imaging modalities and MRI sequences, grouped according to feature class and image type. *GLCM*, gray-level cooccurrence matrix; *GLDM*, gray-level dependence matrix; *GLRLM*, gray-level run length matrix; *GLSZM*, gray-level size zone matrix; *ICC*, intraclass correlation coefficient; *LoG*, Laplacian of Gaussian

**2D**	**Feature class**	**Image type**	**Total features (*n*)**	**Matching features (*n*)**	**Matching features (%)**	**ICC ≥ 0.75 (*n*)**	**ICC ≥ 0.75 (%)**
**CT + MRI (T1w + T2w)**	First order	LoG	72	61	84.72	4	6.56
		Original	18	15	83.33	0	0
		Wavelet	72	45	62.50	3	6.67
	GLCM	LoG	88	74	84.09	2	2.70
		Original	22	11	50.00	0	0
		Wavelet	88	55	62.50	2	3.64
	GLDM	LoG	56	41	73.21	4	9.76
		Original	14	7	50.00	0	0
		Wavelet	56	29	51.79	6	20.69
	GLRLM	LoG	64	48	75.00	1	2.08
		Original	16	10	62.50	1	10.00
		Wavelet	64	36	56.25	7	19.44
	GLSZM	LoG	64	40	62.50	1	2.50
		Original	16	7	43.75	0	0
		Wavelet	64	28	43.75	3	10.71
	Shape	Original	9	8	88.89	4	50.00
	Overall		783	515	65.77	38	7.38
**MRI (T1w + T2w)**	First order	LoG	72	63	87.50	8	12.70
		Original	18	15	83.33	0	0
		Wavelet	72	50	69.44	6	12.00
	GLCM	LoG	88	80	90.91	2	2.50
		Original	22	17	77.27	1	5.88
		Wavelet	88	65	73.86	2	3.08
	GLDM	LoG	56	43	76.79	2	4.65
		Original	14	9	64.29	1	11.11
		Wavelet	56	37	66.07	6	16.22
	GLRLM	LoG	64	51	79.69	1	1.96
		Original	16	12	75.00	2	16.67
		Wavelet	64	46	71.88	2	4.35
	GLSZM	LoG	64	50	78.13	1	2.00
		Original	16	8	50.00	0	0
		Wavelet	64	40	62.50	2	5.00
	Shape	Original	9	9	100.00	4	44.44
	Overall		783	595	75.99	40	6.72

**Table 4:** 3D matching features. Number and percentage of matching stable features obtained with 3D contour-focused segmentation, as well as number and percentage of matching stable features with good (ICC ≥ 0.75) overall interobserver reliability across different imaging modalities and MRI sequences, grouped according to feature class and image type. *GLCM*, gray-level cooccurrence matrix; *GLDM*, gray-level dependence matrix; *GLRLM*, gray-level run length matrix; *GLSZM*, gray-level size zone matrix; *ICC*, intraclass correlation coefficient; *LoG*, Laplacian of Gaussian

**3D**	**Feature class**	**Image type**	**Total features (*n*)**	**Matching features (*n*)**	**Matching features (%)**	**ICC ≥ 0.75 (*n*)**	**ICC ≥ 0.75 (%)**
**CT + MRI (T1w + T2w)**	First order	LoG	72	57	79.17	0	0
		Original	18	10	55.56	0	0
		Wavelet	144	97	67.36	0	0
	GLCM	LoG	88	75	85.23	4	5.33
		Original	22	16	72.73	0	0
		Wavelet	176	147	83.52	6	4.08
	GLDM	LoG	56	37	66.07	5	13.51
		Original	14	8	57.14	0	0
		Wavelet	112	72	64.29	6	8.33
	GLRLM	LoG	64	48	75.00	1	2.08
		Original	16	7	43.75	0	0
		Wavelet	128	81	63.28	3	3.70
	GLSZM	LoG	64	34	53.13	0	0
		Original	16	5	31.25	1	20.00
		Wavelet	128	72	56.25	6	8.33
	Shape	Original	14	12	85.71	11	91.67
	Overall		1132	778	68.73	43	5.53
**MRI (T1w + T2w)**	First order	LoG	72	65	90.28	8	12.31
		Original	18	12	66.67	0	0
		Wavelet	144	116	80.56	14	12.07
	GLCM	LoG	88	75	85.23	10	13.33
		Original	22	16	72.73	2	12.50
		Wavelet	176	149	84.66	16	10.74
	GLDM	LoG	56	42	75.00	6	14.29
		Original	14	9	64.29	1	11.11
		Wavelet	112	83	74.11	10	12.05
	GLRLM	LoG	64	50	78.13	3	6.00
		Original	16	9	56.25	1	11.11
		Wavelet	128	96	75.00	10	10.42
	GLSZM	LoG	64	47	73.44	2	4.26
		Original	16	10	62.50	0	0
		Wavelet	128	92	71.88	6	6.52
	Shape	Original	14	14	100.00	12	85.71
	Overall		1132	885	78.18	101	11.41

Regarding the machine learning pipeline, the number of selected features ranged from 1 (from 2D annotations on T2-weighted images) to 236 (2D annotations on CT images). The accuracy of the ET models was fair to good, ranging between 77% (2D annotations on CT images) and 90% (3D annotations on T2-weighted images). Table 5 reports the results of each annotation and image type combination.

**Table 5:** Feature selection process and exploratory machine learning pipeline in the reproducible feature datasets. The results of each annotation and image type combination are reported

**Annotation type**	**Imaging modality**	**Selected features (***n***)**	**Accuracy (%)**
2D	T1w	5	83
	T2w	1	83
	CT	236	77
3D	T1w	67	87
	T2w	14	90
	CT	108	80

## Discussion

The main finding of our study is that the rates of stable radiomic features extracted from unenhanced CT and MRI were 75% or higher for 2D and 80% or higher for 3D contour-focused segmentation. 3D CT-based texture analysis provided more stable features than 2D approach, while no difference in feature stability rates was found between 2 and 3D MRI-based texture analyses. Overall, a certain degree of segmentation variability highlighted the need to include a reliability analysis in future studies.

Despite its great potential as a non-invasive biomarker to quantify several tumor characteristics, radiomics still faces challenges to clinical implementation, both standalone and paired to machine learning [13, 29]. A great variability in radiomic features has emerged as a major issue across studies, and segmentation is the most critical step [12]. Image segmentation represents the basis of radiomic image analysis pipelines and can be time-consuming if performed manually. Therefore, methodological analyses are advisable prior to conducting radiomic studies in order to assess the robustness of different segmentation approaches and avoid biases due to non-reproducible, noisy features. These analyses have been previously performed in kidney [30, 31], lung, and head and neck [15] lesions. With regard to cartilaginous bone tumors, radiomic studies to date have focused on discriminating among benign, atypical, and malignant lesions [32–35], differentiating chondrosarcoma from other entities such as skull chordoma [36], or predicting recurrence of chondrosarcoma [37]. To our knowledge, our work is the first comprehensively addressing the influence of interobserver manual segmentation variability on the reproducibility of 2D and 3D CT- and MRI-based texture analysis in cartilaginous bone tumors. Nonetheless, Fritz et al. [33] and Gitto et al. [34] performed an interobserver reliability assessment as a feature-reduction method in their radiomic analysis, which provided a model for prediction of tumor grade. In particular, Fritz et al. found that most 2D features derived from unenhanced (15 out of 19) and contrast-enhanced (18 out of 19) T1-weighted MRI had at least good agreement between two observers, using an ICC cutoff of 0.6 [33]. In this study, however, the number of extracted features was only 19 per sequence, the impact of different feature classes was not analyzed, and filtered and transformed images were not used. Despite these issues, a common conclusion that can be drawn from this and our studies is that most MRI radiomic features of cartilaginous bone tumors have good reproducibility, even though a certain degree of segmentation variability exists. In a more recent study by Gitto et al., stability was assessed as a feature-reduction method and CT radiomic features were considered stable if ICC 95% confidence interval lower bound was 0.75 or higher. This resulted in a lower feature stability rate (30%) [34] compared to our current study.

In our study, all imaging modalities demonstrated good reproducibility both employing 2D and 3D annotations, with a robust feature percentage ranging from 75 to 96% for the former and 80 to 95% for the latter. Stable features also proved quite informative for predictive modeling at our preliminary analysis, with accuracies of 77–90%. Given the limited sample size and presence of 3 class labels, this result is promising and supports the use of radiomic data in this research domain. These findings are encouraging for future radiomic analyses, even though they confirm the need for a preliminary assessment of feature stability, and in line with recent literature emphasizing the importance of reproducibility in artificial intelligence and radiology [38]. The higher spatial resolution of CT did not seem to influence feature reproducibility and was probably offset by the better contrast resolution of T1-weighted and T2-weighted images. Furthermore, margin shrinkage did not lead to improvements in terms of feature reproducibility, contrary to a previous investigation on renal cell carcinoma CT images [17]. It should be noted that in this investigation, however, the authors reported that margin shrinkage produced less informative features even with improved reproducibility [17].

We found higher rates of stable features derived from CT for 3D compared to 2D segmentation, but no difference in the rates of 2D and 3D MRI-derived stable features. This finding is in favor of a 2D approach in future radiomic studies dealing with MRI-based texture analysis of cartilaginous bone tumors, as this is less time-consuming and easier to be employed in clinical practice, particularly in large atypical cartilaginous tumors and chondrosarcomas. Furthermore, most 2D (66–76%) and 3D (69–78%) stable features matched between CT and MRI, as well as T1-weghted and T2-weighted images. Finally, shape-based features were stable even across different imaging modalities and MRI sequences, and were thus reproducible and independent descriptors of tumor size and shape. On the other hand, overall interobserver reliability of other feature classes was unsurprisingly low across different imaging modalities and MRI sequences, indicating that their quantitative values depend on the specific image used.

Some limitations of our study should be acknowledged. First, it has a retrospective design as a prospective analysis is not strictly necessary for radiomic studies [13]. The retrospective design accounts for the exclusion of contrast-enhanced images, as they were not performed for all enchondromas. Contrast-enhanced and dynamic contrast-enhanced MRI improve the accuracy of cartilaginous bone tumor assessment [39–41] and future radiomic studies focusing on these sequences are warranted. Finally, due to its scope, this was a single-institution study and generalizability of our findings needs to be confirmed on more varied datasets.

## Conclusions

In conclusion, radiomic features of cartilaginous bone tumors extracted from 2D and 3D segmentations on CT and MRI examinations are reproducible, although some degree of segmentation variability highlights the need to perform a preliminary reliability analysis in radiomic studies. 3D and 2D MRI-based texture analyses provide similar rates of stable features. Thus, a 2D approach can be favored in future studies, as this is easier to implement in clinical practice.

## Abbreviations

2D: Bidimensional
3D: Volumetric
CT: Computed tomography
ET: Extra Trees
GLCM: Gray-level cooccurrence matrix
GLDM: Gray-level dependence matrix
GLRLM: Gray-level run length matrix
GLSZM: Gray-level size zone matrix
ICC: Intraclass correlation coefficient
LoG: Laplacian of Gaussian
MRI: Magnetic resonance imaging
RFE: Recursive feature elimination
ROI: Region of interest
